# Does earlier use of productivity enhancers during cell line selection lead to the identification of more productive cell lines?

**DOI:** 10.1186/1753-6561-5-S8-P9

**Published:** 2011-11-22

**Authors:** Alison J Porter, Atul Mohindra, Juana Maria Porter, Andrew J Racher

**Affiliations:** 1 Lonza Biologics plc, 228 Bath Road, Slough, Berkshire, SL1 4DX, UK

## Background

After selection of a recombinant cell line for the production of a therapeutic protein, attempts are often made to increase volumetric productivity. Various techniques have been employed during the production process when trying to increase product concentration. Some aim to increase the time integral of viable cell concentration (IVC; the number of hours viable cells are available to produce the product) by increasing the maximum viable cell concentration and maintaining high viabilities. Techniques include (i) optimization of media and feeds, (ii) optimization of feeding strategies and (iii) genetic manipulation. Others aim to increase specific production rate (Q_P_) by the deliberate inhibition of cell growth (controlled proliferation). Three methods commonly used to control proliferation are use of (i) chemical agents, (ii) hypothermic conditions and (iii) genetic manipulation. In this study, we focused on increasing volumetric productivity by manipulating Q_P_; in particular, by the use of chemical agents.

As a cell line has typically already been selected as a manufacturing cell line prior to assessing such methods to increase Q_P_, the likelihood of success is not predictable: resulting in the frequently heard comment that results are ‘cell line specific’.

But what if we were to look at these methods with many cell lines, at a much earlier stage of development (before the final cell line has been selected)? It could be that the cell line with the highest product concentration is typically a non-responder.

The work described looks to answer the questions: (1) To what extent does the response to such methods vary in a large panel of cell lines producing the same antibody? and (2) Would their use in an earlier stage of cell line development enable the selection of a ‘better’ manufacturing cell line?

## Materials and methods

Cell lines: A panel of 148 GS-CHO cell lines was generated by transfecting the host cell line CHOK1SV with the GS vector pEE12.4 containing the gene for the model antibody cB72.3 (Porter *et al*, 2010, Biotechnol Prog 26: 1455-1464).

Cell culture: The cell lines were assessed in a scale-down model (fed-batch shake-flask cultures) of Lonza Biologics’ final production bioreactor process, using CDACF medium and feeds. Three cultures were initiated for each cell line. The first was a control culture, in the second the culture medium was supplemented with 1 mM Sodium Butyrate (NaBu), and in the third the culture medium was supplemented with 7.5 mM Sodium Acetate (NaAc). The cell concentration was determined using a Vi-CELL automated cell counter. Product concentration was determined using Protein A HPLC.

## Results

The distribution of the values for the parameters IVC, Q_P_ and product concentration were investigated for each condition (control, NaBu and NaAc). For IVC, the distribution of the values for both the NaBu and NaAc conditions are lower than that of the control. For Q_P_, the distribution of values for the NaBu condition are higher that that of the control. The NaAc condition shows no improvement. For product concentration, the distribution of values for the NaBu condition are lower than that of the control. Little difference is observed between the control and the NaAc condition. Data were analyzed by one-way ANOVA and Tukey’s multiple comparison test at a 5% significance test. The analysis reveals that there is a significant difference between the control and NaBu conditions for IVC, Q_P_ and product concentration. In addition, there is a significant difference between the control and NaAc conditions for IVC but not for Q_P_ and product concentration.

**Figure 1:**
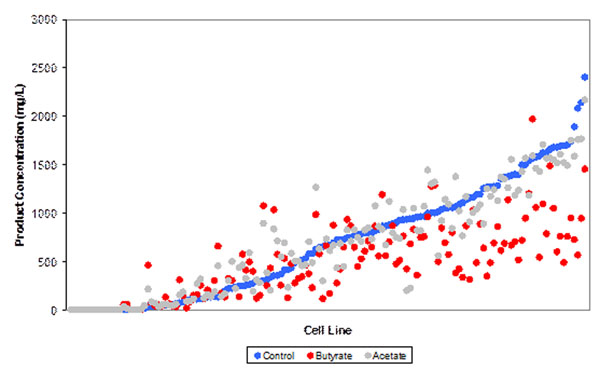
Full distribution of product concentration values for the control, NaBu and NaAc cultures

Review of individual cell lines (Figure 1) reveals that

■ The majority of cell lines did not achieve a higher product concentration compared to the control, when either NaBu or NaAc was added

■ The use of NaBu resulted in an increase in productivity, compared to the control, for 27% of the cell lines

■ The use of NaAc resulted in an increase in productivity, compared to the control, for 41% of the cell lines

■ An increase in productivity was approximately twice as likely to be observed with the use of NaAc compared to the use of NaBu

■ For both NaBu and NaAC, an increase in product concentration compared to the control was more likely if the cell line was a low producer

■ For those cell lines exhibiting an increase in product concentration compared to the control when NaBu or NaAc was added, 80% and 62% respectively were in the lower 50% of producers when ranked by control product concentation

■ The highest product concentration was achieved using control conditions

■ No advantage of NaAc or NaBu if looking to identify a more productive cell line from the population

## Conclusions

The data generated in the rigorous testing support the anecdote that the ability of productivity enhancers to increase Q_P_ is ‘cell line specific’. On average, no increase in mean product concentration was seen and only ~25-50% cell lines exhibited a benefit. The question ‘Would the use of productivity enhancers in an earlier stage of cell line development enable the selection of a ‘better’ manufacturing cell line?’ has been answered: There is no advantage in their use. If the selection strategy has identified a high producing cell line, the productivity enhancers are unlikely to be effective. Also, the highest product concentration was achieved with control conditions.

